# Improvement on Selective Laser Sintering and Post-Processing of Polystyrene

**DOI:** 10.3390/polym11060956

**Published:** 2019-06-01

**Authors:** Zhi Zeng, Xiaohu Deng, Jiangmei Cui, Hai Jiang, Shuo Yan, Bei Peng

**Affiliations:** 1 School of Mechatronics Engineering, University of Electronic Science and Technology of China, Chengdu 611731, China; zhizeng@uestc.edu.cn (Z.Z.); haijiang@uestc.edu.cn (H.J.); 201722080301@std.uestc.edu.cn (S.Y.); beipeng@uestc.edu.cn (B.P.); 2 National Local Joint Engineering Laboratory of Intelligent Manufacturing Oriented Automobile Die & Mold, Tianjin University of Technology and Education, Tianjin 300222, China; 3 School of Materials Engineering, Chengdu Technological University, Chengdu 611730, China; cjmei@cdtu.edu.cn

**Keywords:** selective laser sintering, polystyrene, orthogonal test, post-processing, mechanical properties

## Abstract

Amorphous polymers are heavily utilized materials in selective laser sintering (SLS) due to their good dimensional accuracy. However, sintered parts of amorphous polymers cannot be used as functional parts owing to their poor forming performance, including their low relative densities and tensile strength. Therefore, post-processing methods are employed to enhance the mechanical properties of amorphous polymers SLS parts without damaging their relatively high dimensional accuracy. In this study, the forming process of selective laser sintering (SLS) and post-processing on polystyrene (PS) was investigated. The orthogonal experiment was designed to obtain the optimal combination of process parameters. The effect of a single process parameter and the laser volumetric energy density (LVED) on dimension accuracy and warpage of the sintered parts were also discussed. In addition, a three-dimensional (3D) thermal model was developed to analyze the temperature fields of single-layer SLS parts and PS powder sintering mechanism. Then, infiltrating with epoxy resin was employed to enhance the mechanical properties of the PS parts. Good resin-infiltrated formulation was obtained based on the mechanical property tests and fractured surface analysis. This research provides guidance for SLS process and post-processing technology in polymers.

## 1. Introduction

Selective laser sintering (SLS) is one of the most efficient additive manufacturing techniques, which employs laser scanning to make a three-dimensional part from powder materials. Polymers are heavily utilized materials in SLS because of their thermal properties. However, there are several problems in polymer materials by SLS, including molding dimensional error, strength and post-processing technique. Proper process equipment and experimental conditions may lead to great differences in molding.

Many investigations on sintering molding of polymer have been conducted in recent years. It is important that SLS parts have high accuracy to ensure the functional requirements. Raghunath et al. investigated the relationship between shrinkage and SLS parameters of polyamide and optimum shrinkage conditions were obtained by Taguchi method [1]. Senthilkumaran et al. [2] presented an experimental study to understand the shrinkage in SLS polyamide 12 and the effect of building strategies on shrinkage behavior. Singh et al. [3,4] investigated the correlation between different SLS process parameters and dimensional accuracy by developing mathematical models. The above studies mainly focused on the semicrystalline polymer. Shi et al. [5] investigated the sintering parameters and properties of the sintered parts of high-impact polystyrene and found the specimen had a good accuracy. Recently, Wei et al. [6] investigated the effects of laser power, scanning speed and layer thickness on the dimensional precision and morphology of the polystyrene (PS) parts. It was indicated that the optimum dimensional accuracy could be obtained by suitable process parameters. In addition, warpage is also a key factor in part accuracy in the SLS process. Wang et al. [7] studied the influence of process parameters on warpage when sintering PS in SLS. The results provided a better understanding of the influences of processing parameters on warpage in SLS process.

Besides the sintering part’s accuracy, SLS should be able to provide sufficient mechanical properties to meet the functional requirements. Beal et al. [8] evaluated the effect of the variation of the energy density on the mechanical properties of polyamide. The research results indicated that laser power had more influence on the density and mechanical properties than the scan speed. Yan et al. [9] investigated both the polystyrene and styrene–acrylonitrile copolymer SLS process. The influences of laser energy density on the sintering density, mechanical properties and dimensional accuracy of the SLS parts were studied. Singh et al. [10,11] carried out a set of experimental studies on optimization of shrinkage, mechanical properties and density. The effects of the different process parameters were also analyzed. Zhu et al. [12] analyzed the tensile properties and thermomechanical properties of polypropylene samples fabricated by SLS. Bai et al. [13] performed a study on the feasibility of processing a polyethylene by SLS. The effect of temperature on the mechanical properties of the sintered parts was also discussed. Recently, Pilipovic et al. [14] expanded the process parameters that affect the mechanical properties and the process parameters depending on the manufacturing strategy and layer thickness was confirmed. However, the effects of process parameters on dimensional accuracy and mechanical properties were different.

Above all, the amorphous polymer SLS parts have very low relative density and much lower tensile strength than that of their fully dense forms, while the semicrystalline polymer SLS parts have higher relative density and comparable tensile strength compared to their fully dense forms. Yan et al. [15] studied the difference in the part bed temperature, relative density, tensile strength and dimensional accuracy of the SLS parts between PS and nylon-12 (PA12). They found that the dimensional accuracy of the SLS parts of amorphous polymers was higher than that of semicrystalline polymer SLS parts with the same processing parameters. Therefore, post-processing methods were employed to enhance the mechanical properties of amorphous polymers SLS parts without damaging their relatively high dimensional accuracy. At present, infiltrating with resin or wax is the most commonly used post-processing methods. Shi et al. [16,17,18] applied epoxy resin to improve the mechanical properties of PC SLS parts and found that the mechanical properties were improved remarkably by post-processing.

It is difficult to measure the real time temperature changes, melting zone size and the thickness of solid phase during the SLS process. Therefore, finite element (FE) simulation has been introduced to describe the SLS process. Dai and Shaw [19] investigated the effect of the volume shrinkage during SLS using a three-dimensional finite element model, and indicated that the models with the volume shrinkage provided better prediction in the shape and size of the sintering parts. Dong et al. [20] developed a transient three-dimensional finite element model, which could simulate the temperature and density distribution. The simulated results showed that the sintered depth was dependent on the laser power and scanning speed. Recently, Ganci et al. [21] proposed a numerical approach to model the SLS of polypropylene. In their study, a 3D thermal thermomechanical model was set up to predict both the temperature fields and the distortion of the sintered parts. Lindberg et al. [22] investigated SLS PA12 using FE method. It was found that FE simulations given good estimations for the location of a failure. Mokrane et al. [23] developed a numerical tool for simulating SLS of polymer powders, which was validated at the numerical level and tested against the experimental study. These studies indicate that the FE model can provide good prediction of temperature and mechanical properties evolutions during SLS process.

Polystyrene has become a widely used polymer material for SLS due to its relatively low cost and good process ability [24,25]. However, few systematic studies on the whole molding process and post-processing of PS SLS process were carried out. The relationship between the different printing parameters and part properties can be investigated with application of Taguchi method [26]. In this paper, orthogonal experiment was used to analyze the effects of laser power, scanning speed, layer thickness and scanning interval on sintered parts. Subsequently, the influences of different molding process parameters on the dimension, warpage and sintering density of the sintered parts were discussed. In addition, a FE method was employed to further study temperature evolutions and sintering mechanisms. At last, the infiltrated epoxy resin experiment was presented to investigate the effects of different epoxy resin systems on mechanical properties of the parts.

## 2. Materials and Methods

### 2.1. Materials

**Figure 1:**
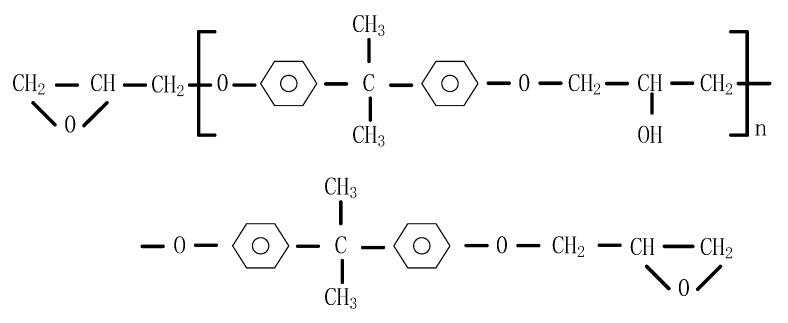
Chemical structure of CYD-128.

The PS powder used in this study was purchased from RTP Company (winona, MN, USA) with the particle size ranging from 75 to 106 μm, which are regarded as the appropriate sizes for SLS. The properties of PS are listed in Table 1. A two-component epoxy resin was synthesized. Component A contained a bisphenol A epoxy resin CYD-128, an epoxide active diluent Alkyl(C12-C14)glycidyl ether (AGE), and curing accelerator 2.4.6-Tri(dimethylaminomethyl)Phenol (DMP-30); component B was a curing agent of T31 (phenolic amine types) or 593 (aliphatic amine types). CYD-128 epoxy resin was provided by Baling Company (Yueyang, China), SINOPEC. T31 Curing agent, 593 curing agent, and DMP-30 curing accelerator were all purchased from Ji’nan sunny Chemical Technology Co., Ltd (Ji’nan, China). AGE diluent was provided by Ji’ning Bai Yi Chemical Co., Ltd (Ji’ning, China). The molecular formula of CYD-128 is shown in Figure 1.

### 2.2. Selective Laser Sintering

SLS of PS powder was performed using the YBRP-360 SLS system (YIBO SANWEI, Beijing, China) equipped with a CO_2_ laser with adjustable fill power ranging from 0 to 30 W. It is generally accepted that pre-heating temperature, laser power, scanning speed, layer thickness and scanning interval exert significant effect on the dimension and warpage. In this study, the preheating temperature was set to 85 °C based on previous research results [6,15], while other parameters were altered at discrete intervals. Optimum process parameters of PS parts were selected by the orthogonal test of four factors and four levels, with the laser power, scanning speed, layer thickness and scanning interval represented as A, B, C and D respectively. The orthogonal factor level table is shown in Table 2. In this study, the effects of the printing parameters on the dimensional accuracy and warpage were analyzed.

### 2.3. Post-Processing

PS parts of SLS are not suitable for the practical production due to their poor mechanical properties. Therefore, post-processing was employed to improve the strength and surface quality. Infiltrating epoxy resin was conducted to investigate the influences of the kinds and proportions of curing agents. The theoretical content of curing agent is dependent on epoxy equivalent of epoxy resin and diluent. The stoichiometric quantity of amine curing agent per 100 g of epoxy resin can be calculated by the following equation [27]

(1) $$\mathrm{Amine}\;\mathrm{equivalent}\;\mathrm{weight}=\frac{\mathrm{MW}\;\mathrm{of}\;\mathrm{amine}}{\mathrm{no}.\mathrm{of}\;\mathrm{active}\;\mathrm{hydrogens}}$$

(2) $$\mathrm{pph}\;\mathrm{of}\;\mathrm{amine}=\frac{\mathrm{amine}\;\mathrm{equivalent}\;\mathrm{weight}\;\times 100}{\mathrm{epoxy}\;\mathrm{equivalent}\;\mathrm{weight}\;\mathrm{of}\;\mathrm{resin}}$$

In addition, temperature has a significant effect on the amount of hydrogen. The proportions of epoxy resin system were designed by the theory calculation, as shown in Table 3. The penetration depth was dependent on the compatibility between PS and epoxy resin, part porosity and viscosity. Before infiltrated resin, the epoxy resin was heated to 30 °C for reducing viscosity.

### 2.4. Measurements

**Figure 2:**
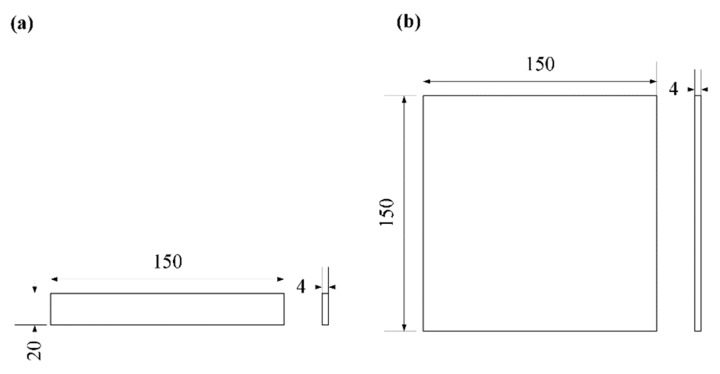
Geometric models of the precision test specimens. (**a**) dimensional accuracy; (**b**) warpage.

Geometric models of the PS parts for dimensional accuracy, warpage test are shown in Figure 2. The dimensional accuracy of the PS SLS specimens is represented with the dimensional error S, which can be calculated by

(3) $$S=\frac{L_{1}-L_{0}}{L_{0}}\times 100\%$$

where $L_{0}$ is the design size, $L_{1}$ is the actual size measured by a vernier caliper.

**Figure 3:**
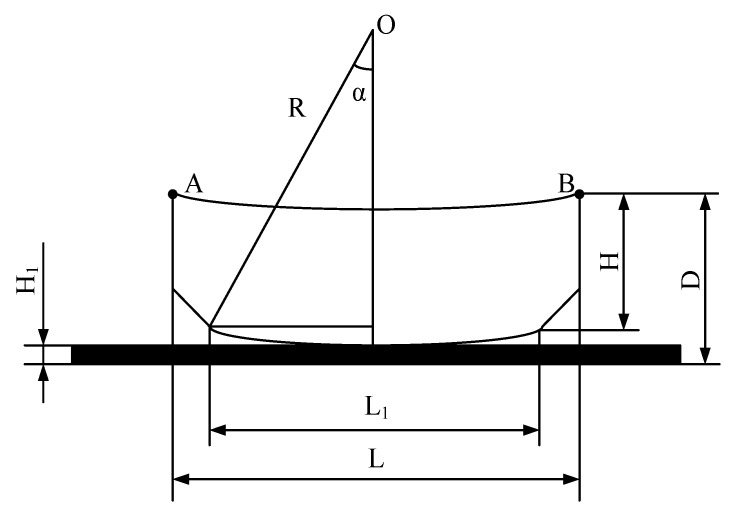
Schematic of warpage calculation.

The dimensions of the specimen for warpage test was 150 × 150 × 4 mm^3^, and the warpage can be calculated by

(4) $$m=(D-H_{1}-H)/L$$

where D is the total thickness, $H_{1}$ is the thickness of the glass plates, H is the thickness of the specimen, L is the height of the specimen. Schematic of warpage calculation can be seen in Figure 3.

The microscopic morphology of the fractured surfaces of PS parts was investigated by a scanning electron microscope (SEM) (VTIEESWC A N VEGA3, Brno, Czech Republic). The specimens were sputter-coated with gold-palladium to avoid charging.

**Figure 4:**
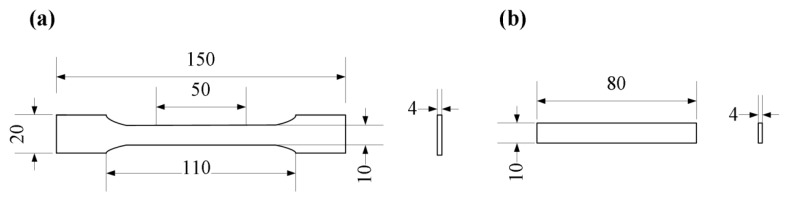
Geometric models of the mechanical test specimens. (**a**) Tensile specimen; (**b**) bending specimen.

The tensile and bending tests were measured on the post-processed PS parts. Tensiling specimens were designed according to ISO 178:2001 with the loading rate of 5 mm/min. The three-point bending tests were performed according to the ISO 527-2:1993 with the loading rate of 2 mm/min. Both the tensile test and three-point flexural test were conducted using the KL-WS-30S universal material experiment machine (KL-WS-30S, Dongguan Kunlun Instrument Co., Ltd., Dongguan, Guangdong, China). The geometric models are shown in Figure 4. For each data point, five specimens were conducted to eliminate the interference of experimental error.

## 3. Results and Discussion

### 3.1. Optimization of Molding Parameters

**Figure 5:**
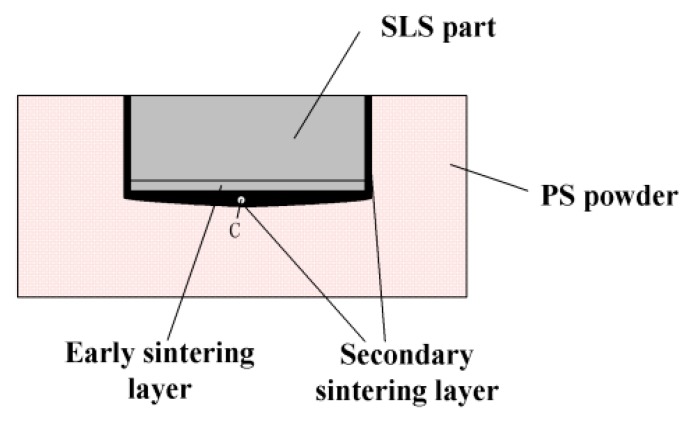
Secondary sintering of SLS part.

The dimension accuracy, warpage for different parameters are listed in Table 4. The dimensional accuracy of X, Y, and Z direction are 1.23 to 1.5%, 1.14 to 3.35%, and −3.4 to −0.75%, respectively. It can be observed that the sizing shrinks in the direction of length and width, while extends in the direction of height. The reasons can be attributed to the secondary sintering caused by the temperature gradient between the sintered part and loose powder around it, as shown in Figure 5. During the continuous sintering, the heat transfer from the upper layer to the lower layer, thermal energy stored in the sintered part propagates outward into the surrounding loose powder and raise local temperatures. When temperature of powder around and at the bottom of the workpiece reaches the glass transition temperature for amorphous polymers, the powder will be adhered to the SLS part and form the irregular surface resulting in an increase in part size. This is in line with previous research that the secondary sintering may cause part growth and thus increase in the dimensions of parts for amorphous polymers [9].

It has been proven that part growth is prone to occur when the laser energy density ω is great, which can be described by

(5) $$\omega =\frac{P}{H\times v}$$

where *P* is the laser power, *H* is the scanning interval, and *v* is the scanning speed. Moreover, warpage ranges from 0.07 to 0.69%. It is evident that warpage is easy to occur in high energy density.

The combined effect of process parameters on the part dimension and performance can be reflected on the role of the laser volumetric energy density (*LVED*), which can be defined by

(6) $$LVED=\frac{P}{v\times H\times t}$$

where t is the layer thickness (mm). There will be interaction between different process parameters, for example, the increase of laser energy can increase parts sintering density and strength, and it may also cause a large temperature gradient and raises the warpage. Therefore, the optimization of laser scanning path should be performed to obtain uniform parts temperature field distribution and reduce the warpage deformation.

**Figure 6:**
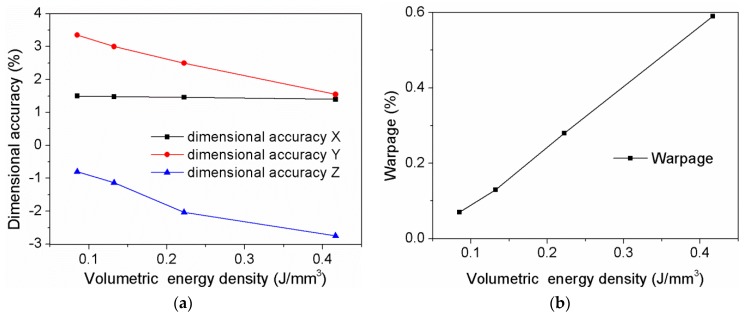
The effects of the laser volumetric energy density variation. (**a**) Dimensional accuracy; (**b**) warpage.

Figure 6 shows the effects of the LVED on the dimensional accuracy and warpage. It can be seen that significant differences are observed among the dimensional errors in the X, Y, and Z directions, and the variation trendency is also distinct. It is indicated that the dimensional errors in X and Y directions are positive, and in the Z direction is presented as negative. Furthermore, the positive error decreases with the increase of the LVED while the negative error increases. It can be attributed to a phenomenon referred to part growth in the SLS process, as shown in Figure 5. The second sintering phenomenon mainly occurs in both bottom and side of the sintered layer, and the secondary sintering effect of the bottom is stronger than side. Therefore, the change of dimensional error in Y and Z directions is significant. Moreover, the larger the LVED, the more obvious part growth. It is confirmed that the warpage enlarge by the increase of the LVED, as shown in Figure 6b.

In order to obtain the optimized process parameters, range analysis was used in the study of the warpage and dimensional accuracy. Range analysis of orthogonal experiments is listed in Table 5 and Table 6, respectively. As shown in Table 5, the optimal combination of warpage is A4B1C4D4, which is corresponding to the laser power of 13.4 W, scanning velocity of 800 mm/s, layer thickness of 0.24 and scanning interval of 0.28, respectively. The sequence according to the warpage is as follows: layer thickness, laser power, scanning interval and scanning velocity.

Table 6 shows the range analysis of the dimensional accuracy. Four level ranges of dimensional accuracy of X direction are largely consistent. It can be seen that the optimal combination of dimensional accuracy of X direction is A2B1C1D2, in which the laser power, scanning velocity, layer thickness and scanning interval is 9.8 W, 800 mm/s, 0.15 and 0.2, respectively. In contrast, four ranges of dimensional accuracy of Y and Z direction have a significant difference. The sequence according to the effect of dimensional accuracy of Y direction is as follows: scanning velocity, laser power, scanning interval and layer thickness. It can be concluded that the optimal combination of elongation is A2B1C1D4, in which the laser power, scanning velocity, layer thickness and scanning interval is 9.8 W, 800 mm/s, 0.15 and 0.28, respectively. Unlike the dimensional accuracy of Y direction, the sequence based on the effect of dimensional accuracy of Z direction is as follows: layer thickness, scanning velocity, laser power and scanning interval. The optimal combination of dimensional accuracy of Z direction is A2B1C4D1, which is 9.8 W in laser power, 800 mm/s in scanning velocity, 0.24 in layer thickness of and 0.16 in scanning interval, respectively.

It can be concluded that the optimal part precision varies with different process parameters. Therefore, the influence of these factors on the precision should be comprehensively considered to determine the optimal SLS process parameters. Factor B and C exerted the greatest effect on the dimensional accuracy of Y and Z direction, respectively. As per the dimensional of height, the major factor of warpage is factor C, which mainly depends on the interactions between the laser power and the laser sintering of powder. On the basis of these factors, the optimal combination of process parameters is C4A2B1D4.

### 3.2. FEM Analysis of PS SLS Process

A finite element model was introduced to simulate the single-layer laser sintering process. The selected process parameters were as follows: laser power of 9.8 W, pre-heating temperature of 85 °C, scanning speed of 800 mm/s, layer thickness of 0.24 mm, scanning spacing of 0.28 mm, and laser spot diameter of 0.2 mm, respectively.

**Figure 7:**
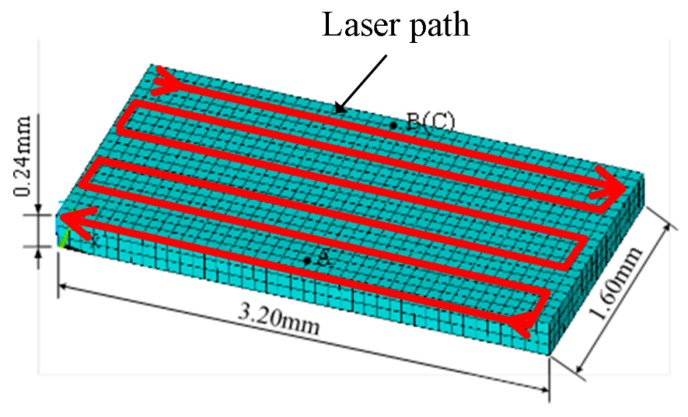
Meshed model.

The numerical model was developed using the FE software ANSYS with reference to Figure 7, and the laser scanning path is also schematically shown in the figure. The heat transfer was set to two boundary conditions. The first condition was defined on the powder bed surface, which accounts for the energy lost through the radiation and the convection. The second condition satisfies the requirement that no heat is lost through the side and bottom of the powder bed. The boundary conditions can be discribed by the following equation

(7) $$-k_{e}\frac{\partial T}{\partial z}{|}_{z=0}+h(T_{s}-T_{ext})+\sigma \varepsilon (T_{s}{}^{4}-T_{ext}^{4})=q$$

where, $k_{e}$ is the heat conductivity coefficient, h is the convection heat transfer coefficient in the enclosed processing box, $T_{s}$ is the temperature, $T_{ext}$ is the environment temperature, σ is the Stefan–Boltzmann constant, and ε is the thermal radiation coefficient. PS powder size was set to 0.08 mm uniform for mesh generation and convergence. The dimensions were 3.2 mm × 1.6 mm × 0.24 mm. The thermal properties of PS powders were achieved by referring to in [23]. The heat source for the SLS process is the laser beam. Gaussian distribution is a well-known function to characterize the laser energy distribution. In the present model, the laser energy density distribution can be described by

(8) $$q(r)=Q_{m}\exp\left(-\frac{2r^{2}}{r_{0}^{2}}\right)$$

where, $r_{0}$ is the laser spot radius, r is the radial distance from the center of the laser beam, $Q_{m}$ is the maximum beam intensity and can be calculated by

(9) $$Q_{m}=\frac{2*A*P}{\pi r_{0}^{2}}$$

where, A is the laser absorptivity, P is the laser power. The thermophysical properties and laser properties are listed in Table 7.

**Figure 8:**
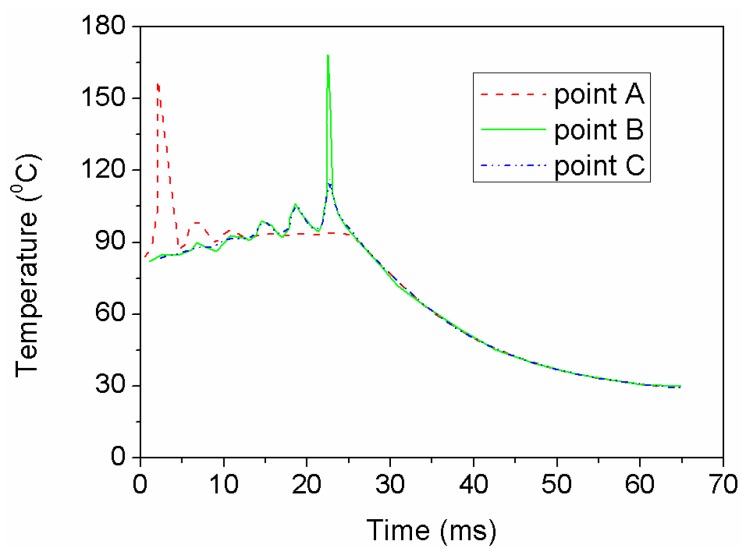
Temperature time history of identification of points.

Three tracking points were identified as shown in Figure 7 (C was positioned in a bottom surface). Figure 8 shows the temperature histories for the considered points. It can be observed that the temperature changes exhibit multiple peaks. The predictions are consistent with those in the literature [21]. For points A, the first peak is generated due to the laser in the first track, and the rest are caused by the overlap of the adjacent position. For the points that in the previous track were in the periphery of the laser, i.e., point B and C, temperature gradually rises, and the maximum temperature comes in the last peak. In addition, temperature of point C is apparently lower than that of point B. A temperature difference between point B and C has a significant effect on the sintering capability between layers. The temperature of powder bed surface decrease dramatically due to the radiation and the convection.

**Figure 9:**
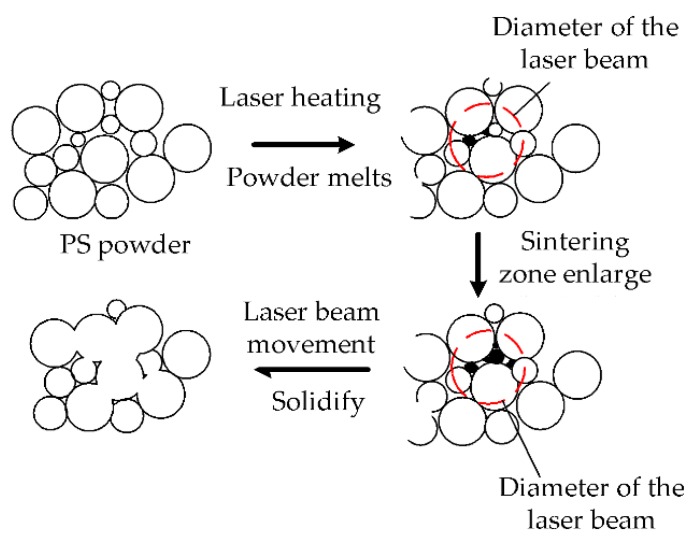
Sintering mechanism of SLS process.

Figure 9 shows the forming mechanism in polymer powder sintering. During the sintering process, the powder at the center of the laser spot reaches the melting point first to form a melting pool rapidly. The process of melting corresponds to the first track, as shown in Figure 7. After the first track, the powder fully solidifies. Then, with the continuous action of the laser energy, the unmelted powder in the zone of action begins to melt and flows to the molten pool. In this process, the molding room has both convection of air and other forms of heat loss. When the laser moves rapidly, the molten pool solidifies with the decrease of heat, and the whole part gradually cooled down. The shrinkage of the part occurs with the decrease of temperature. The reason lies in the powder particles or particle clusters sliding from their initial positions to fill the vacancies. This happens when sinter contacts are thermally activated by the applied temperature, which reduces the friction between the individual particles, causing them to slide [28]. The above phenomenon will repeat until the end of SLS process.

**Figure 10:**
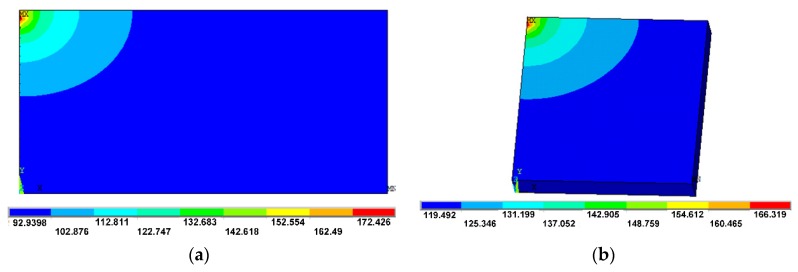
Temperature distribution in different part size (°C). (**a**) Large-size model; (**b**) small-size model.

Figure 10 shows that the part size exerts a great effect on the temperature field. When the dimensions of part are larger, a reciprocating laser scanning time is longer, resulting in the decrease of temperature of the sintered part. At the same time, the preheating effect of adjacent powder begins to reduce, resulting in a large temperature gradient and shrinkage. Figure 10b shows the temperature distribution when the dimension of part lets up the half. Compared to Figure 10a, it can be concluded that the temperature gradient reduces with the decreasing of part dimension. It is helpful to reduce parts warpage and shrinkage.

### 3.3. Post-Processing Parts Properties

**Figure 11:**
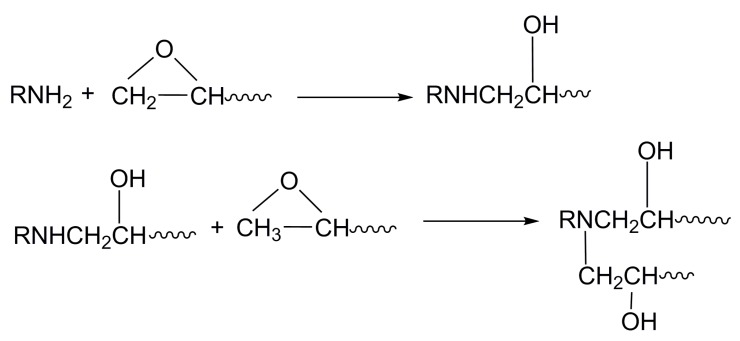
Cure reaction mechanism of amine and epoxide [29].

The tensile and bending specimens were printed at laser power of 9.8 W, pre-heating temperature of 85 °C, scanning speed of 800 mm/s, layer thickness of 0.24 mm, scanning spacing of 0.28 mm, and laser spot diameter of 0.2 mm. The tensile strength and flexural strength of PS SLS specimens are 1.6 MPa and 2.81 MPa, respectively, which cannot meet the need of functional parts or investment casting of thin-walled parts. The SLS parts were infiltrated by two different epoxy resin system. The curing reactions of CYD-128 with amine in the presence of OH groups are shown in Figure 11. Amine-type curing agents react with epoxide rings by nucleophilic addition. The degree of infiltration is dependent on the content and viscosity of the curing agent. The content of curing agent has a significant effect on the infiltrating process. It is indicated that the curing velocity accelerates with the increase of the curing agent content. However, the penetration period prolongs with the increase of curing agent content. An excess of curing agent may result in curing at super-fast velocity and hence the small penetration depth. In the present study, the penetration depth of the most infiltrated parts exceeds 150 mm, which is the maximum size of the tensile specimen. The exceptions are No. 4 and No. 5 parts because of viscosity and the long penetration period.

**Figure 12:**
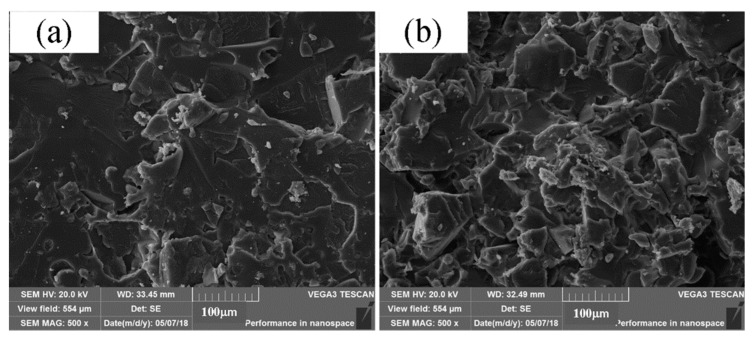
Microstructure of fracture surface. (**a**) Curing agent T31; (**b**) curing agent 593.

Before being infiltrated, the adjacent particles mainly bond together by diffusion, and the PS particles do not melt completely in the SLS process. The link between the particles is connected by forming local sintering necks at only the contact points. The individual particles can still be identified and a large number of pores can be obviously seen [6,15]. Therefore, the microstructure shows less dense and porous, thus the lower strength and fragile. Figure 12 shows the microstructures of the fractured tensile specimens after being treated. After infiltrated with the epoxy resin, it can be seen that the pores among particles are filled with epoxy resin. Then, the epoxy resin will undertake most of the load on specimens and significantly improve the mechanical properties of PS parts. The post-processing parts have high density and strength. Moreover, it is obvious seen that the parts infiltrated by curing agent 593 have more compact structure than those infiltrated by curing agent T31.

**Figure 13:**
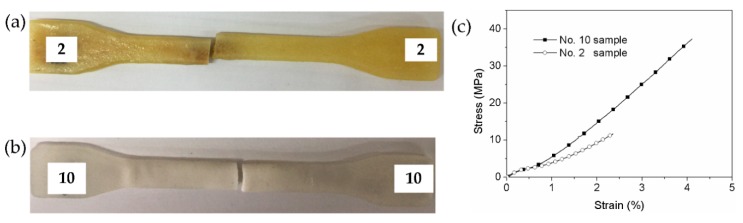
Fractured tensile specimens. (**a**) No. 2 sample; (**b**) No. 10 sample; (**c**) stress–strain curves.

The effect of curing agent on mechanical properties was also investigated. No. 2 and No. 10 specimens were selected to analyze the effect of the epoxy resin system on tensile properties. Figure 13 shows the fractured specimens after the tensile tests. The tensile strength is 11.75 MPa and 36.81 MPa, respectively. In addition, No. 10 specimen presents higher elongation rate than No. 2 specimen. It is indicated that the curing agent 593 can significantly improve the tensile strength and bending strength compared to curing agent T31. The difference may be attributed to the different viscosity of epoxy resin system. It is observed that the viscosity of epoxy resin adding 593 is lower than that by adding T31. Moreover, it is seen that the mechanical properties dramatically reduce with the increasing of T31 contents due to the high viscosity of T31 system and poor permeability of resin. Therefore, epoxy resin is easier to infiltrate SLS parts by adding 593, thus producing better mechanical properties.

Table 8 shows that the measured tensile strength and bending strength for different types and content of curing agents, which corresponds to Table 3. The contents of the curing agent must also have a significant effect on the improvement of mechanical properties. In general, greater strength can be obtained by adding more curing agents. The maximum tensile strength and bending strength are 37.78 MPa and 52.36 MPa, corresponding to the No. 10 parts, which are close to the tensile strength of full density PS, 42.5 MPa.

## 4. Conclusions

The effect of SLS processing parameters on the part quality of PS materials was investigated in this study, especially the dimensional accuracy and warpage. The influences of laser power, scanning speed, layer thickness and scanning interval on the part were analyzed by orthogonal tests. The range analysis indicated that the optimal combination of process parameters was laser power of 9.8 W, scanning speed of 800 mm/s, layer thickness of 0.24 mm and scanning interval of 0.28 mm, respectively.

An FE model was developed to analyze the temperature change and distribution of SLS processing to further understand the sintering mechanism. The results show that the greater laser energy density would produce the structural warpage. As a result of secondary sintering, the dimensional of height and the other direction may change in opposite directions. The numerical results also show that the small part size decreases warpage and shrinkage.

Post-processing methods of infiltrating epoxy resin are able to improve the mechanical properties of PS parts. Two different epoxy systems were selected to infiltrate the PS parts. The tensile and bending tests were conducted to the infiltrated epoxy parts, the results of which show that infiltrating epoxy resin can significantly increase their strength. The 593 epoxy system works better than the T31 epoxy system under the same ratio. The maximum tensile and bending strength were 36.81 Mpa and 52.22 MPa, respectively.

## Figures and Tables

**Table 1:** PS properties parameters.

Properties	Parameters		
Physical properties	Density	Hardness	Shrinkage
	1.04~1.065/g∙m^−3^	M65~90	0.2~0.6
Optical properties	Light transmittance	Haze	Refractive index
	80~90%	3%	1.59
Thermal properties	*T*_g_ (glass transition temperature)	viscous flow transition	Decomposition temperature
	70~98 °C	175~195 °C	>300 °C

**Table 2:** Orthogonal factor level table for SLS of PS.

level	Factor			
	Laser Power A	Scanning Speed B	Layer Thickness C	Scanning Interval D
	(W)	(mm/s)	(mm)	(mm)
1	8	800	0.15	0.16
2	9.8	1000	0.18	0.20
3	11.6	1200	0.21	0.24
4	13.4	1400	0.24	0.28

**Table 3:** The proportions of a two-component epoxy resin.

No.	Proportions				
	CYD-128	AGE	DMP-30	T31	593
	(Expoxy Resin/g)	(Diluent/g)	(Curing Accelerator/g)	(Curing Agent/g)	(Curing Agent/g)
1	100	5	2	20	0
2	100	5	2	25	0
3	100	5	2	30	0
4	100	5	2	35	0
5	100	5	2	40	0
6	100	5	2	0	15
7	100	5	2	0	20
8	100	5	2	0	25
9	100	5	2	0	30
10	100	5	2	0	35

**Table 4:** Forming precision of orthogonal array designed specimens.

Factor No.	A (W)	B (mm/s)	C (mm)	D (mm)	Dimensional Accuracy (%)			Warpage (%)
					Length X	Width Y	Height Z	
1	1	1	1	1	1.40	1.55	−2.75	0.59
2	1	2	2	2	1.46	2.50	−2.03	0.28
3	1	3	3	3	1.48	3.00	−1.13	0.43
4	1	4	4	4	1.50	3.35	−0.80	0.07
5	2	1	2	3	1.33	1.21	−1.45	0.28
6	2	2	1	4	1.32	1.35	−3.08	0.57
7	2	3	4	1	1.37	2.00	−0.78	0.20
8	2	4	3	2	1.32	2.35	−1.00	0.43
9	3	1	3	4	1.25	1.14	−0.75	0.19
10	3	2	4	3	1.46	2.45	−0.80	0.35
11	3	3	1	2	1.23	2.65	−3.23	0.69
12	3	4	2	1	1.41	2.75	−1.60	0.50
13	4	1	4	2	1.24	1.85	−0.93	0.24
14	4	2	3	1	1.49	2.15	−1.35	0.33
15	4	3	2	4	1.39	2.25	−1.98	0.13
16	4	4	1	3	1.24	2.80	−3.40	0.32

**Table 5:** Range analysis of warpage.

Range	A	B	C	D
K1	0.34	0.33	0.54	0.41
K2	0.37	0.38	0.3	0.41
K3	0.43	0.36	0.35	0.35
K4	0.26	0.33	0.22	0.24
R	0.18	0.06	0.33	0.17
Optimum levels	A4	B1	C4	D4
Optimum assembly	A4B1C4D4			
Order of priority	C A D B			

**Table 6:** Range analysis of dimensional accuracy.

Direction	Range	A	B	C	D
X direction	K1	1.46	1.31	1.3	1.42
	K2	1.34	1.43	1.4	1.31
	K3	1.34	1.37	1.39	1.38
	K4	1.34	1.37	1.4	1.37
	R	0.13	0.13	0.1	0.11
	Optimum levels	A2	B1	C1	D2
	Optimum assembly	A2B1C1D2			
	Order of priority	B A D C			
Y direction	K1	2.60	1.44	2.09	2.11
	K2	1.73	2.11	2.18	2.34
	K3	2.25	2.48	2.16	2.37
	K4	2.26	2.81	2.41	2.02
	R	0.87	1.38	0.33	0.34
	Optimum levels	A2	B1	C1	D4
	Optimum assembly	A2B1C1D4			
	Order of priority	B A D C			
Z direction	K1	1.68	1.47	3.11	1.61
	K2	1.58	1.81	1.76	1.79
	K3	1.59	1.78	1.06	1.69
	K4	1.91	1.7	0.83	1.65
	R	0.34	0.34	0.23	0.18
	Optimum levels	A2	B1	C4	D1
	Optimum assembly	A2B1C4D1			
	Order of priority	C B A D			

**Table 7:** Thermophysical and laser properties in FE model.

Parameters	Value
heat conductivity coefficient	0.038 W/(m·K)
convection heat transfer coefficient	6 W/(m^2^·K)
thermal radiation coefficient	0.106 W/(m^2^·K^4^)
laser spot radius	0.002 m
laser absorptivity	0.04

**Table 8:** Effect of curing agent on mechanical properties.

No.	Mechanical Properties	
	Tensile Strength/MPa	Flexural Strength/MPa
1(T31)	6.15 ± 1.4	19.23 ± 0.65
2(T31)	10.83 ± 1.49	34.76 ± 1.37
3(T31)	9.03 ± 0.92	31.47 ± 1.41
4(T31)	4.41 ± 0.12	16.12 ± 0.45
5(T31)	1.41 ± 0.20	14.09 ± 0.76
6(593)	10.82 ± 1.06	22.67 ± 1.26
7(593)	30.01 ± 1.11	31.12 ± 1.13
8(593)	31.15 ± 1.14	44.24 ± 0.43
9(593)	32.07 ± 1.73	46.62 ± 2.19
10(593)	37.78 ± 0.42	52.36 ± 1.39
